# Stage-Specific Expression of TNFα Regulates Bad/Bid-Mediated Apoptosis and RIP1/ROS-Mediated Secondary Necrosis in Birnavirus-Infected Fish Cells

**DOI:** 10.1371/journal.pone.0016740

**Published:** 2011-02-03

**Authors:** Wei-Lun Wang, Jiann-Ruey Hong, Gen-Hwa Lin, Wangta Liu, Hong-Yi Gong, Ming-Wei Lu, Ching-Chun Lin, Jen-Leih Wu

**Affiliations:** 1 Institute of Fisheries Science, National Taiwan University, Taipei, Taiwan, Republic of China; 2 Institute of Cellular and Organismic Biology, Academia Sinica, Taipei, Taiwan, Republic of China; 3 Institute of Biotechnology, National Cheng Kung University, Tainan, Taiwan, Republic of China; 4 Department of Aquaculture, National Taiwan Ocean University, Keelung, Taiwan, Republic of China; University of Cambridge, United Kingdom

## Abstract

Infectious pancreatic necrosis virus (IPNV) can induce Bad-mediated apoptosis followed by secondary necrosis in fish cells, but it is not known how these two types of cell death are regulated by IPNV. We found that IPNV infection can regulate Bad/Bid-mediated apoptotic and Rip1/ROS-mediated necrotic death pathways via the up-regulation of TNFα in zebrafish ZF4 cells. Using a DNA microarray and quantitative RT-PCR analyses, two major subsets of differentially expressed genes were characterized, including the innate immune response gene *TNFα* and the pro-apoptotic genes *Bad* and *Bid*. In the early replication stage (0–6 h post-infection, or p.i.), we observed that the pro-inflammatory cytokine TNFα underwent a rapid six-fold induction. Then, during the early-middle replication stages (6–12 h p.i.), TNFα level was eight-fold induction and the pro-apoptotic Bcl-2 family members Bad and Bid were up-regulated. Furthermore, specific inhibitors of TNFα expression (AG-126 or TNFα-specific siRNA) were used to block apoptotic and necrotic death signaling during the early or early-middle stages of IPNV infection. Inhibition of TNFα expression dramatically reduced the Bad/Bid-mediated apoptotic and Rip1/ROS-mediated necrotic cell death pathways and rescued host cell viability. Moreover, we used Rip1-specific inhibitors (Nec-1 and Rip1-specific siRNA) to block Rip1 expression. The Rip1/ROS-mediated secondary necrotic pathway appeared to be reduced in IPNV-infected fish cells during the middle-late stage of infection (12–18 h p.i.). Taken together, our results indicate that IPNV triggers two death pathways via up-stream induction of the pro-inflammatory cytokine TNFα, and these results may provide new insights into the pathogenesis of RNA viruses.

## Introduction

Infectious pancreatic necrosis virus (IPNV) is an aquatic virus that causes acute contagious diseases in freshwater and marine fish, which can result in heavy losses to the aquaculture industry. IPNV is a member of the *Birnaviridae* family [1]. Birnaviruses contain two genome segments (A and B) of double-stranded RNA contained within an unenveloped, medium-sized, icosahedral capsid [2]. The birnavirus genome encodes three to five structural proteins that are generated through various posttranslational cleavages. VP1 is a viral polymerase that is encoded by the smaller segment, B [3]. The larger segment, A, encodes a polyprotein that is processed into the capsid proteins VP2 and VP3 as well as the viral protease VP4 [4]. Another, smaller open reading frame (ORF) on segment A encodes one 17-kDa non-structural protein, VP5 [5], which is a viral Bcl-2 (B-cell CLL/lymphoma 2) family member that can regulate Mcl-1 and viral protein expression to inhibit apoptosis of infected cells [6], [7].

Two main types of cell death can be easily distinguished: apoptosis and necrosis [8], [9], [10]. Apoptotic cell death is a physiological event that is important during the development and maintenance of tissues. Apoptosis is an active and energy-conserving form of cell death that eradicates aged or diseased cells and poses little threat to the organism. Indeed, it does not lead to activation of the immune system but rather results in the quick clearance of the dying cells by phagocytes without the concomitant induction of an inflammatory response. In contrast, cell death induced by other means, such as injury, leads to necrosis, a form of non-programmed and destructive cell death. Necrosis is characterized by the disturbance of energy metabolism, disruption of cellular membranes, and release of cytoplasmic and nuclear components into the extracellular environment. However, it has become clear that necrotic cell death is as tightly controlled as caspase-dependent apoptosis, and it may be an important mode of cell death that is both pathologically and physiologically relevant [11], [12].

TNFα (tumor necrosis factor alpha) is a pro-inflammatory cytokine that plays important roles in diverse host responses, including cell proliferation, differentiation, necrosis, apoptosis, and the induction of other cytokines. TNFα can induce either NF-κB-mediated survival or apoptosis depending on the cellular context [13]. TNFα mediates powerful anti-microbial responses, including the induction of apoptosis, the killing of infected cells, the inhibition of intracellular pathogen replication, and the up-regulation of diverse host response genes. Many viruses have evolved strategies to neutralize TNF by direct binding and inhibition of the ligand or its receptor or modulation of various downstream signaling events [14]. Furthermore, TNF receptor-1 (TNFR1) has been shown to initiate necrotic cell death [15], and TNFα and other cytokines that bind to receptors of different classes have been reported to lead to the generation of ROS (reactive oxygen species) that function as second messengers in the necrotic cell death pathway [16]. In a recent study investigating the molecular mechanisms regulating necrosis, Sato *et al.* identified the gene expression profile induced in mouse mammary FM3A tumors, which required gene expression to trigger necrosis following treatment with an anticancer agent, 5-fluoro-2′-deoxyuridine [17]. TNFα activates the RIP1 kinase-mediated signaling cascade that is necessary for the induction of downstream genes influencing necrosis or apoptosis [18], [19].

Previous studies have shown that IPNV infection induces both apoptosis and secondary necrosis both in a fish cell line [20], [21] and *in vivo* [22]. IPNV infection can trigger the tyrosine kinase-mediated death pathway to induce the pro-apoptotic protein Bad [23], which may act via NF-κB [24]. Then, IPNV can down-regulate the survival factor Mcl-1 [7] and induce MMP (mitochondrial membrane permeabilization), which is blocked by the ANT (adenine nucleotide translocator) inhibitor BKA [25]. Furthermore, IPNV infection can also cause the activation of caspase-9 and -3 [26]. Finally, the submajor capsid protein VP3 can trigger cell death in fish cells [27].

In this study, we examine how IPNV-induced apoptotic cell death is linked to secondary necrosis in the zebrafish cell line ZF4. We used zebrafish oligo-microarray and real-time RT-PCR assays to screen the IPNV-induced cell death-associated gene expression profiles in zebrafish embryonic cells. Early in replication in IPNV-infected cells, the pro-inflammatory cytokine TNFα was up-regulated up to six-fold relative to the negative control. Furthermore, we demonstrate that the IPNV-mediated up-regulation of TNFα regulates both the Bad/Bid-mediated apoptotic pathway and the RIP1 (receptor-interacting protein-1)/ROS-mediated secondary necrosis pathway.

## Results

### IPNV-induced gene expression profiles in zebrafish embryonic cells

We used the zebrafish embryonic cell line ZF4 as a model system to screen IPNV-induced transcriptomes. We first determined if IPNV (multiplicity of infection (MOI) = 5) can infect ZF4 cells. The IPNV replication stages can be divided into early (6 h post-infection (p.i.)), middle (12 h p.i.), and late replication stages (24 h p.i.) in the ZF4 cell system. As seen in Figure S1A, the viral protein VP2 could be detected in IPNV-infected ZF4 cells at 6, 9, and 12 h p.i. Next, ZF4 cells were infected with different viral doses, and cell death was monitored using a viability assay (Figure S1B).

**Figure 1:**
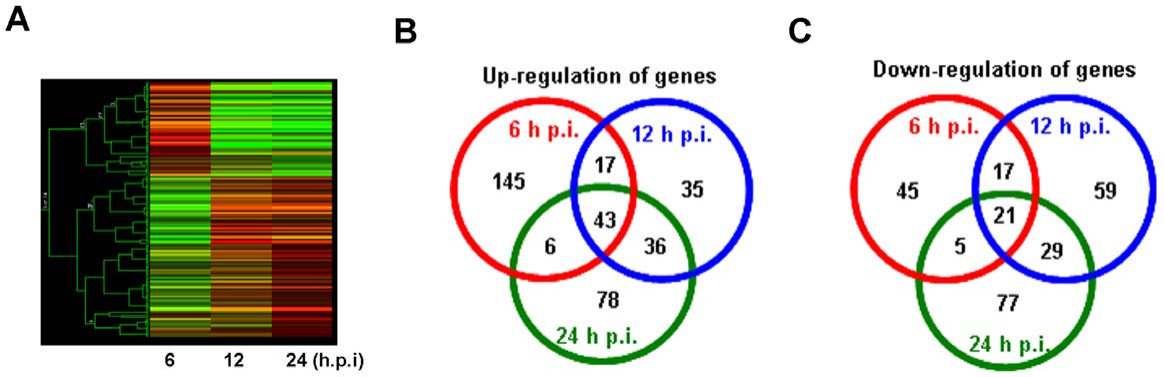
Transcriptional profile of IPNV-infected ZF4 cells 6, 12, and 24 h p.i. (**A**) Hierarchical clustering of the mRNA expression pattern, analyzed using DNA microarrays, compared with the 0 h timepoint. Red indicates up-regulation of genes, green indicates down-regulation of genes, and black is used to represent no change in expression. (**B**) Venn diagram detailing the number of genes up-regulated at 6, 12, and 24 h p.i. in each of three independent experiments examining IPNV-infected ZF4 cells. (**C**) Venn diagram detailing the number of genes down-regulated at 6, 12, and 24 h p.i. in each of three independent experiments examining IPNV-infected ZF4 cells.

We then used the ZF4 cells to analyze the gene expression profile of IPNV-infected cells. The cells were infected with IPNV (MOI = 5), and total RNA was isolated from infected and uninfected control cells at 0, 6, 12, and 24 h p.i. The zebrafish 14K oligo microarray we used comprised 1800 zebrafish gene sequences from the NCBI and a database of 12,768 putative open reading frames derived from NCBI zebrafish EST (expressed sequence tag) sequence information. Overall, the gene expression pattern seen in cells 12 h p.i. was similar to the expression pattern observed at 24 h p.i. (Figure 1A). Furthermore, the expression of genes that were differentially expressed at 6 h p.i. was significantly different from the expression at both 12 h p.i. and 24 h p.i.

Student's *t*-test was used to identify the genes with significant changes in expression relative to the control. We identified 299 transcripts [211 up-regulated (Figure 1B) and 88 down-regulated (Figure 1C)] that demonstrated at least a two-fold change in expression at 6 h p.i. Furthermore, using the same two-fold threshold, 258 (132 up-regulated and 126 down-regulated) and 295 (163 up-regulated and 132 down-regulated) transcripts were differentially regulated at 12 and 24 h p.i. (Figure 1B–C). Table S1 lists the number of significantly (*p*<0.05) differentially expressed genes (greater than two-fold change with respect to the control cells). These transcripts that were significantly modulated following IPNV infection were divided into twelve functional categories: immune response, apoptosis, transcription, signal transduction, lipid and cholesterol metabolism, carbohydrate metabolism, oxidative phosphorylation, cell cycle, protein degradation, protein folding and stress response, protein synthesis, nucleoside metabolism and synthesis. Quantitative real-time RT-PCR was used to confirm the transcriptional changes in select genes. Five up-regulated genes (*mmp9*, *isgf3g*, *bcl-xl*, *cebpb*, and *tnfa*) and three down-regulated genes (*lpl*, *jun*, and *hsp47*) were analyzed, using the expression of *ef1a* as an internal control (Table S2). The real-time RT-PCR data confirmed the same relative transcriptional regulation of the selected genes.

The DNA array data were confirmed using RT-PCR. The expression of the pro-apoptotic genes *bad*, *bmf1*, *bmf2*, *noxa*, and *bax* (Figure S2A) was up-regulated at 6 h p.i. At 12 h p.i., the up-regulation of pro-apoptotic genes *bid*, *puma*, *bok*, and *bok2* (Figure S2B) was analyzed.

### Blockade of TNFα-mediated death signals enhances host cell viability

**Figure 2:**
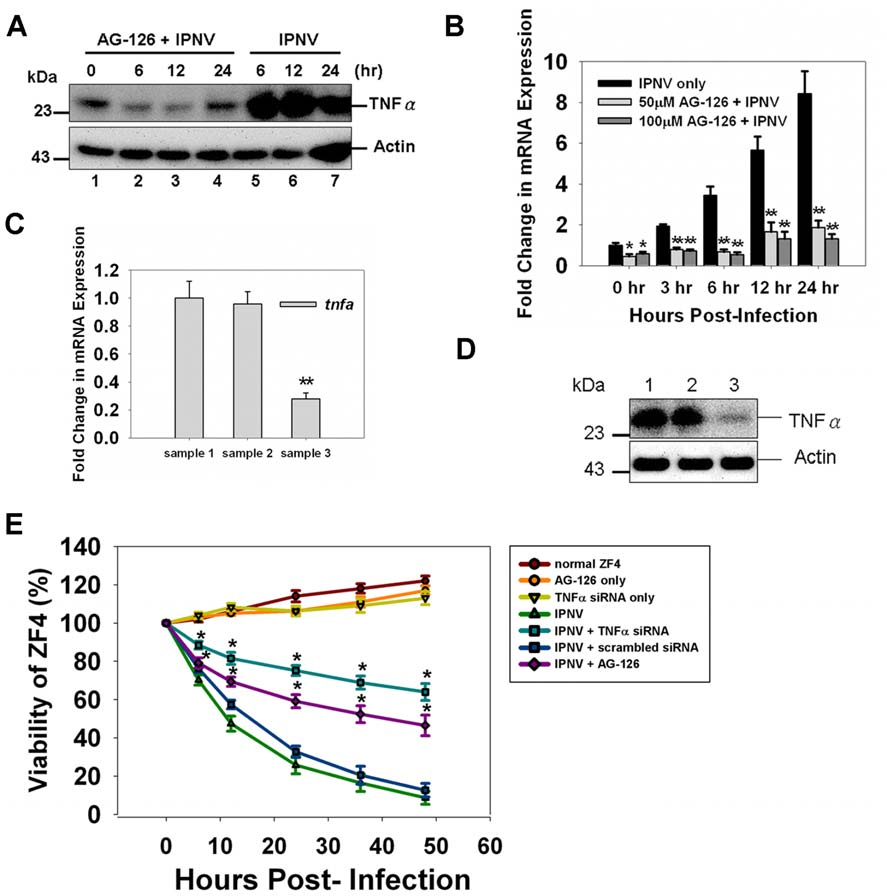
Treatment with TNFα-specific siRNA or AG-126 blocks TNFα expression by IPNV-infected ZF4 cells. (**A**) TNFα protein expression level in IPNV-infected ZF4 cells (MOI = 1) 0, 6, 12, and 24 h p.i. The protein was detected using western blot with a polyclonal antibody specific for TNFα. Lanes 1–4: ZF4 cells were pretreated with 50 µM AG-126 and infected with IPNV for 0 (lane 1), 6 (lane 2), 12 (lane 3), or 24 (lane 4). Lanes 5–7: Untreated ZF4 cells were infected with IPNV for 6 (lane 5), 12 (lane 6), or 24 h (lane 7). The expression of actin was used as an internal control. (**B**) TNFα mRNA expression in IPNV-infected ZF4 cells was quantified using RT-PCR. The ZF4 cells were pre-treated with 50 µM or 100 µM AG-126 for 2 hours, infected with IPNV (MOI = 1), and incubated for 0, 3, 6, 12, or 24 h. The expression of *ef1a* (elongation factor 1-alpha) was used as an internal control. (**C**) The *tnfa* expression was inhibited by TNFα-specific siRNA in IPNV-infected cells. TNFα expression was efficiently inhibited by TNFα-specific siRNA after IPNV infection. Sample 1: ZF4 cells infected by IPNV. Sample 2: ZF4 cells pretreated with scrambled siRNA and then infected by IPNV. Sample 3: ZF4 cells pretreated with TNFα-specific siRNA and then infected by IPNV. The quantification of gene expression in normal versus siRNA-treated cells was calculated relative to *ef1a*. (**D**) Detection of TNFα in untreated or TNFα-specific siRNA-treated ZF4 cells after IPNV infection by western blotting. Lane 1: untreated ZF4 cells; lane 2: ZF4 cells treated with control siRNA; lane 3: ZF4 cells treated with TNFα-specific siRNA. The expression of actin was used as an internal control. (**E**) Cell viability of IPNV-infected ZF4 cells pre-treated with TNFα-specific siRNA or AG-126 at 0, 6, 12, 24, 36 and 48 h p.i. The viability of each sample was determined in three individual experiments. Data shown are the mean ± SD. Student's *t* tests indicate significant differences compared to IPNV infection only or untreated control: *, *p*<0.05; **, *p*<0.01.

We used Pathway Studio 6.0 to search for genes that showed a two-fold or greater expression change in the cDNA microarray and quantitative RT-PCR experiments to see whether TNFα may directly regulate some of the genes (Figure S3). We hypothesized that TNFα plays a crucial role in regulating either the apoptotic or necrotic cell death pathway at different replication stages. TNFα production was specifically inhibited using tyrphostin AG-126, a compound that inhibits the activity of the tyrosine kinases necessary for TNFα production [28]. Following treatment with 50 µM AG-126, the expression of *tnfa* was reduced six-fold (at 6 h p.i., Figure 2A, lane 5), eight-fold (12 h, lane 6) and four-fold (24 h, lane 7) when compared with the untreated IPNV-infected cell (lanes 2–6; 6, 12, and 24 h p.i., respectively). The western blot results were confirmed using real-time RT-PCR, and similar results were obtained. Following treatment with either 50 µM or 100 µM AG-126, the *tnfa* expression level was reduced approximately 10-fold at the 6, 12, and 24 h p.i. timepoints (Figure 2B). We also used RNA interference to investigate whether knocking down TNFα would affect IPNV pathogenesis. The transcriptional expression of *tnfα* was reduced to 25.6% after TNFα-specific siRNA treatment in IPNV-infected cell (Figure 2C). The expression level of TNFα protein was also significantly decreased following siRNA treatment in IPNV-infected cell (Figure 2D).

In addition, the treatment of IPNV-infected cells (MOI = 5) with 50 µM AG-126 increased their viability up to 21.8% (at 12 h p.i.), 33.4% (24 h), and 37.8% (48 h) relative to untreated, IPNV-infected cells. Treatment of IPNV-infected cells (MOI = 5) with TNFα-specific siRNA increased their viability up to 33.8% (at 12 h p.i.), 49.5% (24 h), and 55.3% (48 h) relative to untreated, IPNV-infected cells. Both mock-infected cells and AG-126-treated, uninfected cells were used as negative controls (Figure 2E).

### Recognition of a TNFα-mediated death signal may regulate the expression of the pro-apoptotic genes *bad* and *bid*

**Figure 3:**
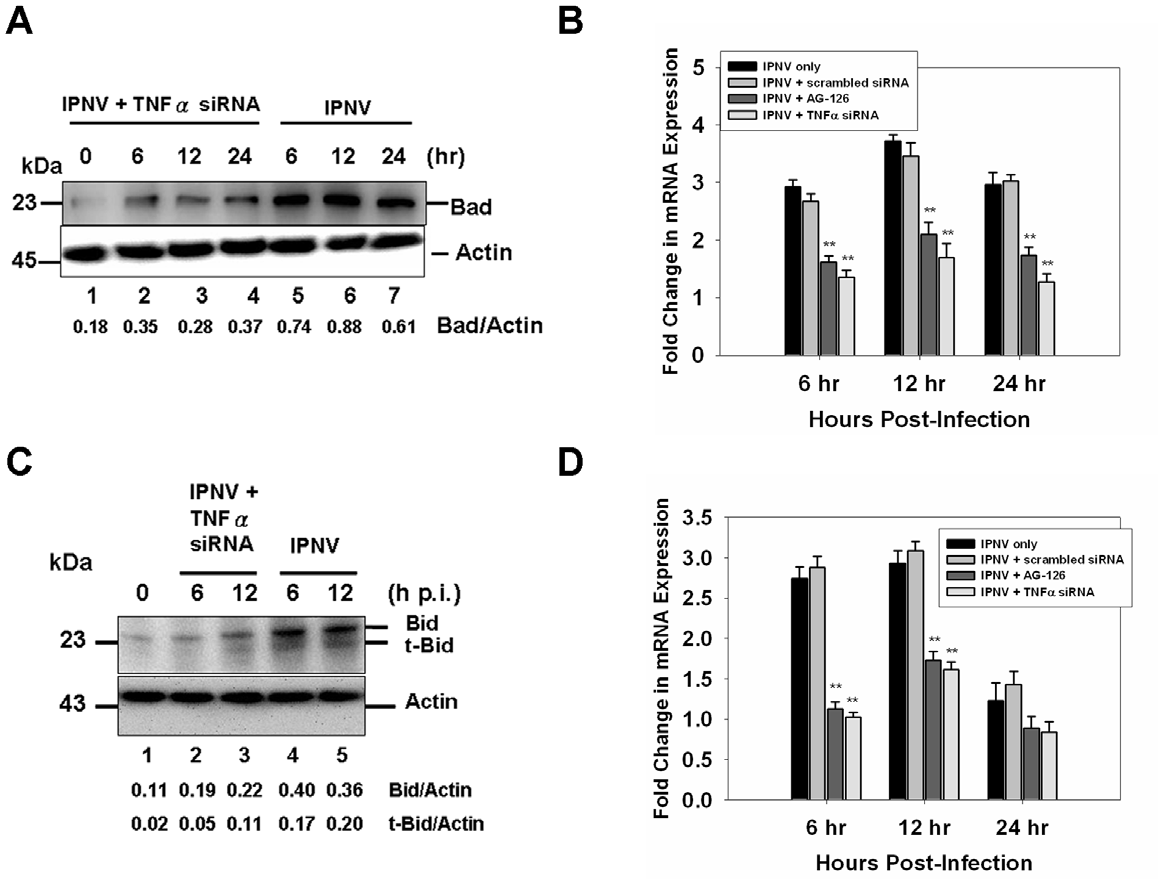
A TNFα-mediated death signal regulates the expression of pro-apoptotic Bad and Bid in IPNV-infected ZF4 cells during the early-middle stage of replication. (**A**) The expression level of the pro-apoptotic proteins Bad and Bid in IPNV-infected ZF4 cells (MOI = 1) at 0, 6, 12, and 24 h p.i. was determined. The proteins were detected using western blot with a polyclonal antibody specific for mouse Bad. Lanes 1–4: ZF4 cells were pretreated with TNFα-specific siRNA and infected with IPNV for 0 (lane 1), 6 (lane 2), 12 (lane 3), or 24 h (lane 4). Lanes 5–7: untreated ZF4 cells were infected with IPNV for 6 (lane 5), 12 (lane 6), or 24 h (lane 7). The expression of actin was used as an internal control. Results are expressed as the ratio of Bad/actin. The mRNA expression of *bad* (**B**) and *bid* (**D**) in IPNV-infected ZF4 cells was quantified using quantitative RT-PCR. ZF4 cells were pre-treated with TNFα-specific siRNA or AG-126 and infected with IPNV (MOI = 1) for 0, 6, 12, or 24 h. The expression of *ef1a* was used as an internal control. Data shown are mean ± SD. Student's *t* tests indicate significant differences compared to untreated control: **, *p*<0.01. (**C**) The expression level of the pro-apoptotic proteins Bid and t-Bid in IPNV-infected ZF4 cells (MOI = 1) at 0, 6 and 12 h p.i. was determined. The proteins were detected using western blot with a polyclonal antibody specific for Bid. Lanes 2–3: ZF4 cells were pretreated with TNFα-specific siRNA and infected with IPNV for 6 (lane 2) or 12 h (lane 3). Untreated ZF4 cells were infected with IPNV for 6 (lane 4) or 12 h (lane 5). Untreated ZF4 cells were infected with IPNV for 0 h (Lane 1). The expression of actin was used as an internal control. Results are expressed as the ratio of Bid/actin or t-Bid/actin.

As seen in Figure 3, inhibition of the IPNV infection-induced TNFα up-regulation reduced the expression of the pro-apoptotic gene Bad by TNFα-specific siRNA at 6 h p.i. (six-fold, Figure 3A, lane 5), 12 h p.i. (eight-fold, lane 6), and 24 h p.i. (four-fold, lane 7) when compared with untreated, IPNV-infected cells (Figure 3A, lanes 1–4). These results were confirmed using quantitative RT-PCR (Figure 3B). Expression of Bid and formation of truncated-Bid were suppressed by TNFα-specific siRNA at 6 h (Figure 3C, lane 2) and 12 h p.i. (lane 3) when compared with the untreated IPNV-infected group (Figure 3C, lanes 4 and 5) and the negative control (0 h, lane 1). The transcriptional expression of *bid* was suppressed after pre-treated with TNFα-specific siRNA or AG-126 in the IPNV-infected ZF4 cells (Figure 3D).

### A TNFα-mediated death signal may induce apoptosis and activate caspases

**Figure 4:**
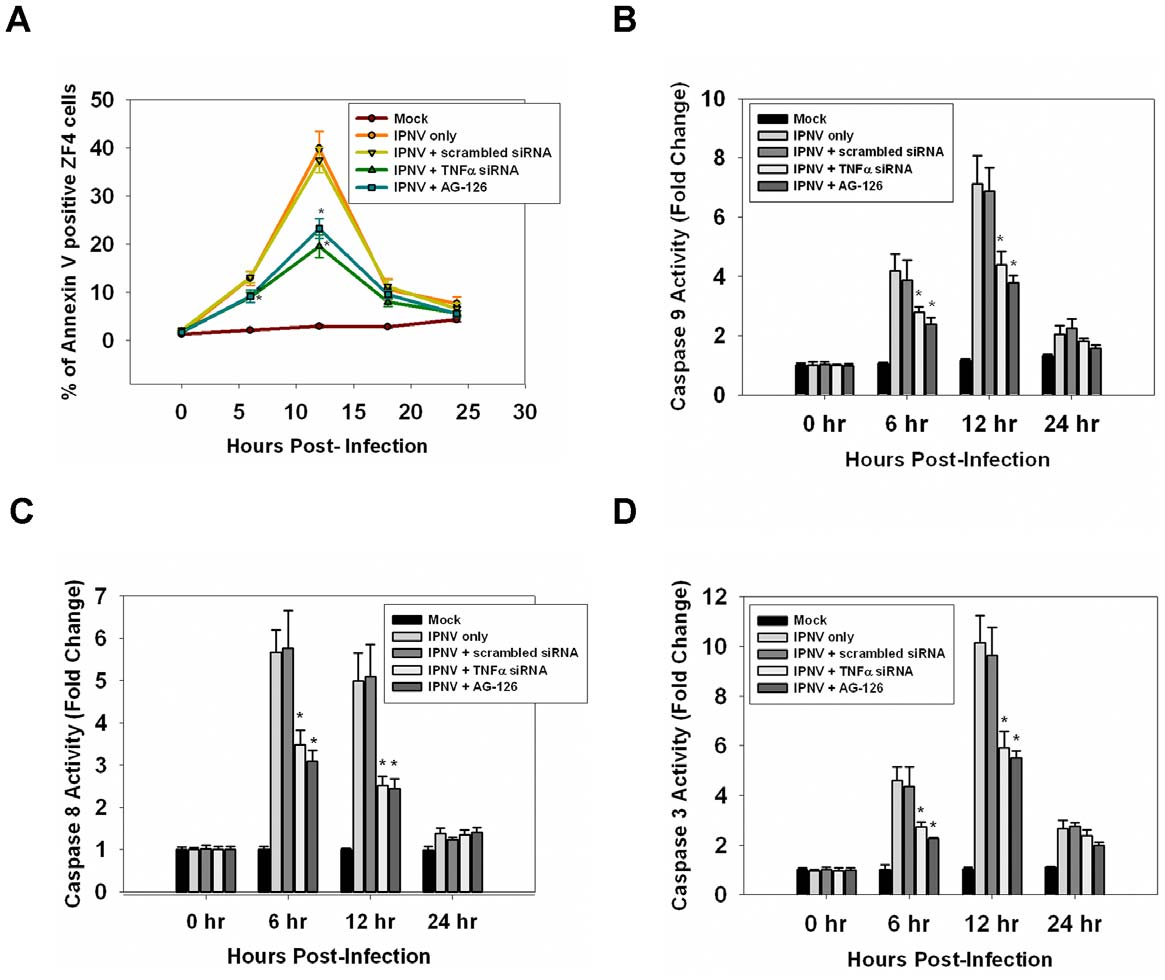
Inhibition of TNFα production can reduce caspase activation and the number of annexin V-positive IPNV-infected cells. (**A**) Detection of annexin V-positive cells following infection with IPNV. ZF4 cells were pre-treated with TNFα-specific siRNA or AG-126, infected with IPNV (MOI = 1), and incubated for 0, 6, 12, 18 and 24 h. Three individual experiments were performed for each sample. (**B**–**D**) Caspase-9, -8, and -3 activities were analyzed. ZF4 cells were pre-treated with TNFα-specific siRNA or AG-126 for 2 hours, infected with IPNV (MOI = 1), and incubated for 0, 6, 12, or 24 h. Luminogenic substrate assays were performed in triplicate. Data shown are the mean ± SD. Student's *t* tests indicate significant differences compared to IPNV infection only: *, *p*<0.05.

Treatment with either AG-126 (50 µM) or TNFα-specific siRNA (20 nM) can reduce the number of apoptotic, annexin V-positive cells by up to 5.2% and 25% (Figure 4A) at 6 h and 12 h p.i. when compared with the IPNV-infected group and mock-infected group. IPNV infection induced dramatic caspase-9 activation at 6 h and 12 h p.i. (Figure 4B), which was only partially blocked by inhibiting TNFα expression. Treatment with TNFα-specific siRNA or AG-126 resulted in a two-fold and three-fold decrease in the number of apoptotic cells at 6 h and 12 h p.i., respectively. Caspase-8 was also activated at 6 h p.i. (5.4-fold) and 12 h (4.7-fold), and this activation was blocked by TNFα-specific siRNA or AG-126 treatment (only a twofold activation was observed) (Figure 4C). Caspase-3 was also activated at 6 and 12 h p.i., and this activation was reduced two- and three-fold, respectively, following treatment with TNFα-specific siRNA or AG-126 (Figure 4D).

### A TNFα-mediated death signal may regulate the apoptotic and secondary necrotic death pathway during the middle stage of replication

**Figure 5:**
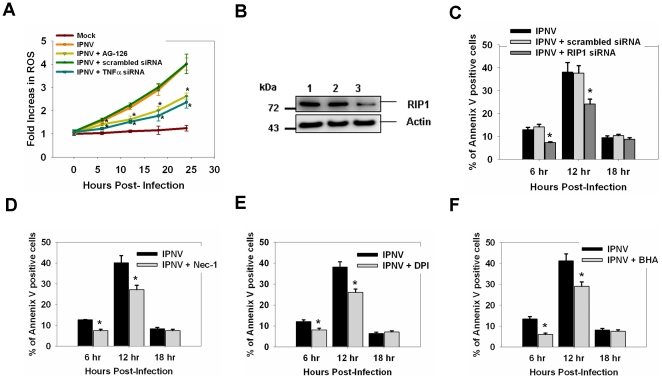
Inhibition of TNFR1 necrotic signaling complex formation inhibits apoptosis and ROS formation in IPNV-infected cells. (**A**) Detection of ROS production in TNFα-specific siRNA or AG-126 pre-treated cells after IPNV infection at 0, 6, 12, 18 or 24 h p.i. Fluorescence assays were performed in triplicate. Determination of the percentage of PI-positive cells after IPNV infection. (**B**) Detection of RIP1 in untreated or TNFα-specific siRNA-treated ZF4 cells by western blotting. Lane 1: untreated IPNV-infected ZF4 cells; lane 2: IPNV-infected ZF4 cells treated with scrambled siRNA; lane 3: IPNV-infected ZF4 cells treated with RIP1-specific siRNA. The expression of actin was used as an internal control. Detection of annexin V-positive cells following infection with IPNV. ZF4 cells were pre-treated with RIP1-specific siRNA (**C**), Nec-1 (**D**), DPI (**E**) or BHA (**F**), infected with IPNV (MOI = 1), and incubated for 0, 6, 12, 18 and 24 h. Three individual experiments were performed for each sample. Data shown are the mean ± SD. Student's *t* tests indicate significant differences compared to IPNV infection only: *, *p*<0.05.

IPNV infection (MOI = 5) caused a 2.1-fold, 3.0-fold, and 4.1-fold increase in ROS production in ZF4 cells 12, 18, and 24 h p.i., respectively. TNFα-specific siRNA (20 nM)- or AG-126 (50 µM)-treated cells displayed a reduction in ROS production at 12 h (1.5-fold), 18 h (1.8-fold) and 24 h p.i. (2.5-fold) (Figure 5A). RNA interference was used to investigate whether knocking down RIP1 would affect IPNV-induced necrosis. The expression level of RIP1 protein was decreased following duplex siRNA treatment (Figure 5B). Following infection with IPNV, the percentages of annexin V-positive, apoptotic cells, were 12.9%, 38.2%, and 9.2% at 6, 12, and 18 h p.i., respectively. Treatment with RIP1-specific siRNA reduced the percentage of PI-positive cells to 7.1%, 24.2%, and 8.4% at 6, 12, and 18 h p.i., respectively (Figure 5C); similar results were seen following treatment with RIP1 inhibitor necrostatin-1 (Nec-1) [29], [30] (Figure 5D). Nox1 is a NADPH oxidase that interacts with Rac1 to promote ROS formation [31], [32]. TNF-induced necroptosis is delayed by inhibition of Nox1 activity [32]. BHA (butylated hydroxyanisole) is a type of anti-oxidant that efficiently inhibits TNF-induced necrotic cell death [25]. The percentages of annexin V-positive cells were reduced after BHA or DPI (diphenylene iodonium, an inhibitor of Nox1) treatment in IPNV-infected cells (Figure 5E–F).

**Figure 6:**
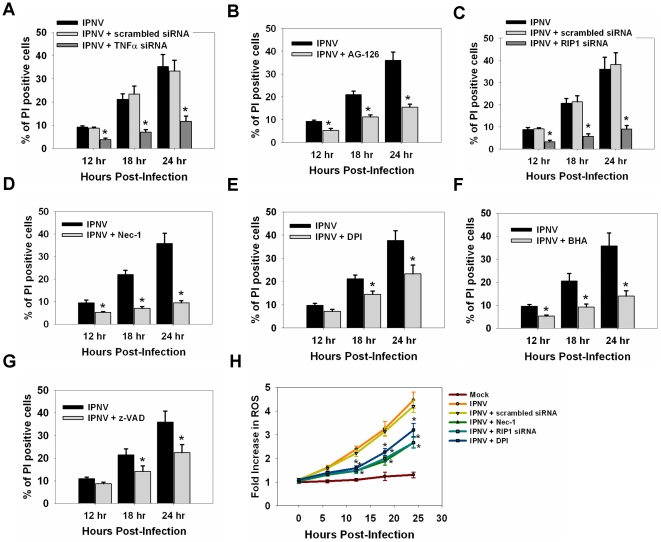
Inhibition of TNF-α or TNFR1 necrotic signaling complex formation inhibits secondary necrosis in IPNV-infected cells. Detection of PI-positive cells following infection with IPNV. ZF4 cells were pre-treated with TNFα-specific siRNA (**A**), AG-126 (**B**), RIP1-specific siRNA (**C**), Nec-1 (**D**), DPI (**E**), BHA (**F**) and z-VAD (**G**) then infected with IPNV (MOI = 5), and incubated for 12, 18 and 24 h. (**H**) Detection of ROS production in RIP1-specific siRNA-, Nec-1- or DPI-pre-treated cells after IPNV infection at 0, 6, 12, 18 or 24 h p.i. Fluorescence assays were performed in triplicate. Data shown are the mean ± SD. Student's *t* tests indicate significant differences compared to IPNV infection only or untreated control: *, *p*<0.05.

Following infection with IPNV, the percentages of PI-positive, necrotic cells, detected using a necrotic death assay, were 9.2%, 21.2%, and 36.5% at 12, 18, and 24 h p.i., respectively. Treatment with TNFα-specific siRNA reduced the percentage of PI-positive cells to 3.8%, 7.0%, and 11.7% at 12, 18, and 24 h p.i., respectively (Figure 6A); similar results were seen following treatment with 50 µM AG-126 (Figure 6B). Recent research has suggested that RIP1 is required for triggering the ROS-mediated necrotic death pathway [33]. Treatment with RIP1-specific siRNA reduced the percentage of PI-positive cells to 3.3%, 5.8% and 9.0% at 12, 18 and 24 h p.i., respectively (Figure 6C). After treating with RIP1 inhibitor Nec-1, the percentage of PI-positive cells was reduced to 3.3% (12 h p.i.), 6.0% (18 h p.i.) and 9.5% (24 h p.i.) (Figure 6D). Treatment with DPI resulted in a reduction in the percentage of PI-positive cells to 7.2%, 14.5% and 23.3% at 12, 18 and 24 h p.i., respectively (Figure 6E). The percentage of PI-positive cells was reduced to 5.3% (12 h p.i.), 9.2% (18 h p.i.) and 14.0% (24 h p.i.) after BHA treatment in IPNV-infected ZF4 cells (Figure 6F). Cells treated with caspase inhibitor (zVAD-fmk) also showed a reduction in the ratio of PI-positive cells after IPNV infection (Figure 6G).

To determine if ROS production is activated through the formation of TNFR necrotic signaling complex, cells were pre-treated with RIP1-specific siRNA, Nec-1 or DPI and then infected by IPNV. ROS production was increased in IPNV-infected cells at 12 (2.1-fold), 18 (3.1-fold), and 24 h p.i. (4.3-fold) (Figure 6H). RIP1-specific siRNA (20 nM) or Nec-1 (20 µM) treated cells displayed a reduction in ROS production at 12 h (1.4-fold), 18 h (1.9-fold) and 24 h p.i. (2.7-fold). Pre-treating ZF4 cells with DPI caused a 1.6-fold, 2.3-fold, and 3.1-fold increase in ROS production 12, 18, and 24 h p.i., respectively.

## Discussion

IPNV causes acute contagious diseases in aquaculture. Therefore, designing effective control or preventive measures against this virus is important. However, an understanding of the mechanisms underlying infection and immunity is essential. In this study, using a zebrafish cell line system, we demonstrated that IPNV regulates the apoptotic and necrotic death pathways through the up-regulation of TNFα. Thus, this study provides new insights into IPNV-induced molecular pathogenesis.

### The zebrafish as a model animal system to examine pathogen-induced transcriptomes

Zebrafish have been recognized and established as a model animal of infectious disease and are considered to have great potential for studying the development and function of the vertebrate immune system. Zebrafish have been shown to be susceptible to infection with and to allow the subsequent replication of various bacterial and viral pathogens. Zebrafish have the advantages of real-time visualization and genetic screens compared to other animal models of infection [34], [35]. IPNV is able to persist and possibly replicate in adult zebrafish [36]. IPNV also activates caspases and promotes host cell apoptosis in a zebrafish cell line [26]. Therefore, zebrafish could be an efficient disease model of IPNV. In our system, we first established the DNA array screening system in ZF4 cells to better understand the virus-host interaction. Our results provide information on the virus as well as alterations in the expression of host genes related to immunity, apoptosis, transcription regulation, unfolded protein response, protein degradation, and the metabolism of cholesterol and carbohydrates following infection with IPNV (Figure S2 and Table S1).

### TNFα-mediated apoptosis and necrosis pathway

TNFα is a crucial regulator in the innate and adaptive immune response against microbial infection via regulation of cell death and survival [37]. TNF is a pro-inflammatory cytokine that plays an important role in diverse host responses such as cell proliferation, differentiation, necrosis, apoptosis, and the induction of other cytokines. Recently, it has been shown that TNF can induce either an NF-κB-mediated survival or apoptotic pathway depending on the cellular context [33]. Many viruses have strategies to neutralize TNF by direct binding and inhibition of the ligand or its receptor or modulation of the various downstream signaling events [14], [38].

The death receptors, including TNFR1, Fas, death receptor 3, DR4, DR5, and the TRAIL receptors, contain an intracellular “death domain” that activates downstream signaling pathways by means of homotypic interactions with adaptor proteins, such as FADD, TRADD, and RIP1 [39]. These death receptors induce apoptosis in many cell types through the activation of caspase-8. Activated caspase-8 may act indirectly to induce apoptosis through cleavage of Bid. Truncated Bid acts on the mitochondria to cause the release of cytochrome *c*, which further activates caspase-9.

TNFR1 has been shown to initiate necrotic cell death [15]. TNFα and other cytokines that bind to receptors of different classes have been reported to induce the formation of ROS that function as second messengers in the necrotic cell death pathway [40], [41]. RIP1 is an intracellular adaptor molecule with kinase activity [42]. RIP1 [19] and RIP3 [43] appear to be crucial in the signaling through cell death receptors that do use caspases to induce death. RIP1 is also necessary for the generation of ROS by TNFα [40], [41]. RIP1 is required for apoptotic death induced by TNFα [44]. IPNV infection of fish cells results in an initial wave of apoptosis that is followed by a second wave of necrosis [20]. In this study, we propose that IPNV induced TNFα up-regulation early in replication when it plays an important role in controlling apoptotic and necrotic cell death [18]. IPNV infection can up-regulate pro-apoptotic genes during the early-middle replication stage, as seen in Figure S2, and this up-regulation can be suppressed by blocking production of TNFα (Figure 3). Furthermore, blockade of TNF production can reduce the apoptotic ratio (Figure 4A) and caspase-3, -8, and -9 activities (Figure 4B–D). During the middle-late replication stage, the TNFα-mediated death signal triggers the necrotic death pathway via the subsequent ROS production (Figure 5A), which is a novel death signal pathway in the zebrafish cell system.

The formation of TNFR1 signaling complex including RIP1, TRADD, Nox1, NOXA1 and Rac1 (small GTPase) is induced by TNF [45], [46]. The kinase activity of RIP1 is essential only for signaling to necrosis [29]. RIP1 is also necessary for the ROS generation by TNFα [42], [47]. Both superoxide generation and cell death in response to TNFα are prevented by knockdown of Nox1 [45]. The activation of Nox1 is downstream of the mitochondrial ROS production [32]. In our study, inhibition of TNFα activation suppressed ROS production in the pathogenesis of IPNV. Inhibition of TNFα, RIP1, Nox1 or generation of ROS could decrease the percentage of annenix V-positive and PI-positive cells after IPNV infection (Figure 5–6). Inhibition of activities of the TNFR1 necrotic signaling complex also suppressed ROS production after IPNV infection. IPNV-induced necrosis might require the formation of TNFR1 necrotic signaling complex. Inhibition of caspase activation was also affected by necrosis induced by IPNV (Figure 6G).

**Figure 7:**
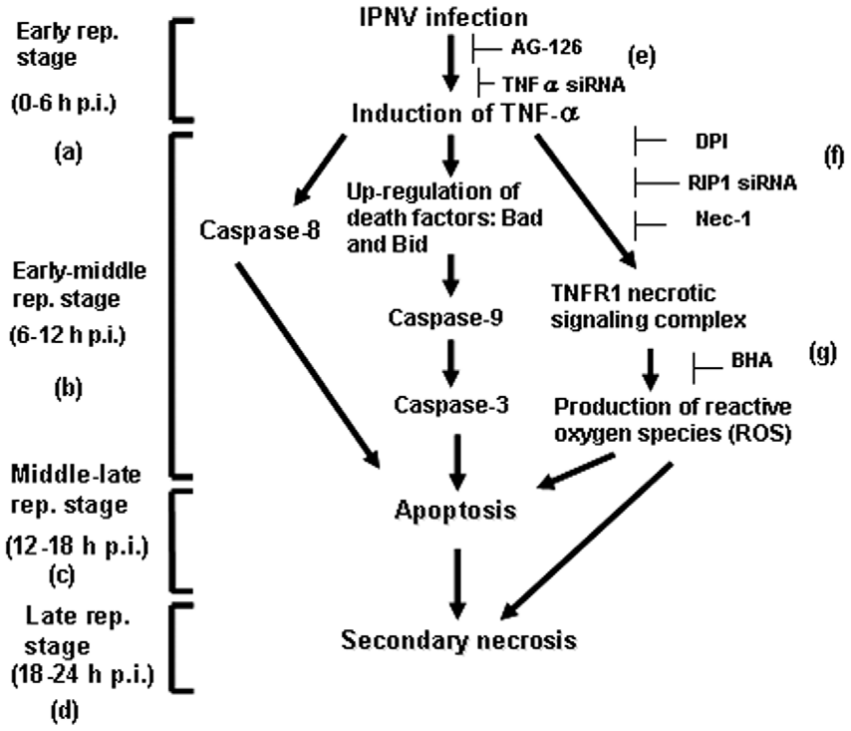
IPNV induces apoptotic and necrotic death cascades via TNFα induction. A hypothesis of how pro-inflammatory TNFα is up-regulated by IPNV infection and how it regulates the apoptotic and necrotic death pathways. When a cell is infected with IPNV (E1-S), the virus binds to the cellular receptor, penetrates the cell, uncoats (the entry stage), and up-regulates the expression of TNFα (a) during the early replication stage (0–6 h p.i.). This up-regulation of TNFα regulates the next wave of gene expression, including the pro-apoptotic genes *bad* and *bid*, during the early-middle stage of replication (6–12 h p.i.; b), at which point the TNFR necrotic signaling complex and reactive oxygen species (ROS) are produced. During this stage, phosphatidylserine (PS) is externalized and endonuclease is released from the mitochondria, resulting in DNA restructuring and cleavage. Furthermore, the cell finally enters the post-apoptotic, necrotic stage during the middle-late stage of replication (12–18 h p.i.; c). During the late replication stage (18–24 h p.i.; d), the cells are broken down. The TNFα-mediated death signal is halted by treatment with a specific inhibitor of TNFα production. (e) TNFα-specific siRNA or AG-126, which block both the pro-apoptotic Bad/Bid-mediated death pathway and the TNFR/ROS-mediated secondary necrotic death pathway. (f) DPI, Nec-1 or RIP-1-specific siRNA, which block the activity of RIP-1 or Nox1 and block the formation of the TNFR necrotic signaling complex. (g) BHA blocks the formation of ROS.

In summary, as shown in Figure 7, we demonstrate that IPNV-induced caspase-mediated, apoptosis and RIP1/ROS-mediated, secondary necrosis requires a TNFα-triggered death signal. Our study may provide new insight into RNA viral pathogenesis.

## Materials and Methods

### Cells, viruses and reagents

The zebrafish ZF4 cell line, which was originally derived from 24-hpf (24 hours post-fertilization) zebrafish embryos, was purchased from the American Type Culture Collection (CRL-2050) and cultured in RPMI 1640 medium supplemented with 10% (v/v) fetal bovine serum and penicillin/streptomycin. The isolated virus, E1-S, a member of the Ab strain of IPNV, was isolated from Japanese eels in Taiwan. The E1-S virus was propagated on a ZF4 cell monolayer at a multiplicity of infection (MOI) of 0.01. Infected cultures were incubated at 18°C until extensive cytopathogenic effects were observed [1]. The virus plaque assays [48] and TCID_50_ were performed on a confluent monolayer of ZF4 cells.

Benzyloxycarbonyl-Val-Ala-Asp (Ome) fluoromethylketone (zVAD-fmk; Promega, USA) was used at 10 µM. 5-(Indol-3-ylmethyl)-(2-thio-3-methyl) hydantoin (Nec-1), BHA, DPI chloride and AG-126 (Sigma Aldrich, USA) were used at 20, 100, 25 and 50 µM, respectively.

### Cell viability assays

For the cell viability assays, the cells were infected with IPNV or pre-treated with AG-126 (50 µM) or TNFα-specific siRNA (20nM) and divided into virus- or mock-infected groups. Cell viability was measured using a colorimetric assay based on the cleavage of the tetrazolium salt WST-1 by mitochondrial dehydrogenase (Cell Proliferation Reagent WST-1; Roche, USA).

### Western blot

Approximately 10^5^ ZF4 cells/ml were seeded in a 60-mm Petri dish (Nunc, Denmark) and cultured for more than 24 h. These cells were then infected with IPNV at an MOI of 5 and incubated for 0, 6, 12, or 24 h. At the completion of each incubation period, the culture medium was aspirated, and the cells were washed with PBS and lysed in 0.5 ml lysis buffer (10 mM Tris base, 20% glycerol, 10 mM SDS, 2% β-ME, pH 6.8). Proteins present in the cell lysate were separated using SDS-PAGE, electro-blotted and subjected to immunodetection as described by Kain *et al.* [49]. The blots were incubated with a 1∶1500 dilution of monoclonal antibody specific for IPNV VP2 (Microtek, Canada), Bad (BD Biosciences, USA), Bid (Millipore, USA), RIP1 (Abcam, UK) or Actin (Millipore, USA) and a 1∶50,000 dilution of a peroxidase-conjugated goat anti-mouse or anti-rabbit antibody (Sigma Aldrich). Alternatively, the blots were incubated with a 1∶3000 dilution of a polyclonal anti-TNFα antibody (AnaSpec, USA) and a 1∶50,000 dilution of a peroxidase-conjugated goat anti-rabbit antibody (Sigma Aldrich). Chemiluminescence detection was performed according to the instructions provided with the Western Exposure Chemiluminescence Kit (GE Healthcare, USA). Resulting western blots were scanned with an imaging densitometer (LAS-3000; Fujifilm, Japan), and optical densities of specific proteins were analyzed with Image Gauge software (Fujifilm).

### Annexin V-FITC labeling

An analysis of phosphatidylserine on the outer leaflet of apoptotic cell membranes was performed using the Annexin-V-FLUOS staining kit (Roche), which contains annexin V-fluorescein and propidium iodide (PI) to differentiate apoptotic cells from necrotic cells. At the end of the various incubation times (0, 6, 12, 18 and 24 h), each sample was removed from the medium and washed with PBS. The cells were incubated with staining solution for 10–15 min. Apoptosis was detected using fluorescence microscopy (Olympus IX70, Japan) with 488-nm excitation and a 525-nm filter for detection [20]. Necrosis was detected using fluorescence microscopy (Olympus IX70) with 535-nm excitation and a 620-nm filter for detection. Each sample group was counted three times, with at least 300 cells counted each time. The mean of the three counts for each different group was used to calculate the apoptotic and necrotic cell indices and their respective standard error.

### RNA preparation

Approximately 10^5^ ZF4 cells/ml were seeded in a 100-mm Petri dish (Nunc) and cultured for more than 24 h. These cells were then infected with IPNV at an MOI of 5 and incubated for 0, 6, 12, or 24 h. At the completion of each incubation period, the culture medium was aspirated, and the cells were washed with PBS. Total RNA was extracted using TRIzol (Invitrogen) and was further purified using an on-column RNase-free DNase digestion (QIAGEN, Germany) to remove possible genomic DNA contamination. The RIN value of the RNA samples before being applied to the microarray was measured using an Agilent 2100 Bioanalyzer (USA) and was 10.0.

### Microarray preparation

The zebrafish 14K oligo microarray comprising 14,067 zebrafish oligonucleotides was designed and synthesized by MWG Genomic Company (Germany) based on 1800 zebrafish gene sequences from the NCBI and a database of 12,768 putative ORFs using NCBI zebrafish EST sequence information [50]. The zebrafish 14K 50-mer oligos were printed on an UltraGAPS Coated Slide (Corning, USA) using an OmniGrid 100 microarrayer (Genomic Solutions, Ann Arbor, USA) according to the manufacturer's instructions. After printing, the slides were baked at 80°C for 6 h, incubated in a glass chamber for 45 min at 42°C in pre-warmed block solution (4× SSC, 0.5% SDS, 1% BSA), quickly washed with distilled water at room temperature, and dipped in room temperature isopropanol. The slides were dried by brief centrifugation.

### Microarray hybridization

Amino-allay dye coupling was carried out using the SuperScript Plus Indirect cDNA Labeling System (Invitrogen) according to the manufacturer's instructions. We optimized the reverse transcription labeling protocol to use 40 µg total RNA, 5 µg anchored oligo (dT) primer ((dT)20VN), and SuperScript III reverse transcriptase. After a 3-h incubation at 46°C, the reaction was stopped by incubating the reaction at 70°C for 15 min in the presence of 1 N NaOH; the solution was then neutralized by adding 1 N HCl. The reaction mixture was brought to a final volume of 100 µl with nuclease-free water. The amine-modified DNA was purified using a MinElute PCR Purification Kit (QIAGEN), following the instructions in the kit, except that the washes were performed twice instead of once and the probe was eluted with 0.1 M NaHCO_3_. One aliquot of Alexa Fluor Dye was then resuspended in 20 µl of aa-cDNA labeled probe and incubated at RT in the dark overnight. The MinElute PCR Purification was then repeated using EB buffer for the elution. Yeast tRNA (10 µg) was added to the sample, which was then dried in a speedvac at 45°C and redissolved in 70 µl of formamide-based hybridization buffer (MWG, Germany). The mixture was then denatured at 95°C for 2 min. The solution was collected by a brief centrifugation and applied onto the oligo area on the microarray slides. Coverslips (60×22 mm) were applied, and the slides were then placed in a chamber and immersed in a water bath for hybridization overnight at 42°C. The arrays were sequentially washed with 2× SSC/0.1% SDS, 1× SSC/0.1% SDS, 0.5× SSC, and 0.1× SSC at room temperature for 5 min per wash. The slides were subsequently dried by brief centrifugation. The arrays were scanned using an Axon GenePix 4000B scanner, and the median spot intensity was determined using an Axon GenePix Pro5.1 (Molecular Devices, Sunnyvale, USA).

### Microarray data analyses

The data files were imported into GeneSpring GX 7.3 (Agilent Technologies, Foster City, USA) for further analysis. “LOWESS Normalization” was applied for data normalization in GeneSpring. The expression data sets must have passed the following quality control categories before they were used for cluster analysis: 1) the hybridization results were not flagged as bad; 2) the net intensity of both channels was equal to or greater than 500; and 3) the statistical analyses were applied to the triplicate data for each spot and repeated three times. For expression data, ratios equal to or greater than 2 were considered up- or down-regulated. DNA microarray data sets were deposited at NCBI's Gene Omnibus Express under the accession number GSE21077. All of the DNA microarray data is MIAME compliant.

### Quantitative real-time PCR

The primers used for quantitative PCR were designed using Primer Express 2.0 software (Applied Biosystems, USA) and are listed in Table S3. For real-time quantitative PCR, first-strand cDNA from ZF4 cells was synthesized using the High Capacity cDNA Reverse Transcription Kit (Applied Biosystems) with random primers. Quantitative PCR was performed using the Power SYBR Green PCR Master Mix (Applied Biosystems) and an ABI Prism 7000 Sequence Detection System.

### Knockdown of TNFα and RIP1 by RNA interference

Duplex small interfering RNA (siRNA) that specifically targeted the mRNA encoding TNFα (BC124141) or RIP1 (BC163762) and scrambled siRNA were commercially synthesized (Sigma, Singapore). The sequences of scrambled siRNA and duplex siRNA that specifically targeted TNFα or RIP1 are listed in Table 1. Duplex siRNAs (20 nM) were transfected into ZF4 cells that were cultured in 60 mm-diameter plastic tissue culture plates (Nunc) using the GeneMute siRNA transfection reagent (SignaGen Laboratories, USA). After a 6-h incubation period, 1 ml RPMI 1640 culture medium containing 10% FBS (Invitrogen) was added to each well without removing the transfection reagent. The cells were then infected with IPNV at an MOI of 1 in 10% FBS RPMI 1640 at 18°C.

**Table 1:** Primer sequences for duplex siRNA that specifically targeted the mRNA encoding TNFα or RIP1 and scrambled siRNA.

Oligo Name	Sequence 5′→3′	Oligo Name	Sequence 5′→3′
*tnfa*-siRNA-sense	CUCUGAAUCAAAGACCUUA	*tnfa*-siRNA- antisense	UAAGGUCUUUGAUUCAGAG
*rip1-*siRNA-sense	CAAAUGCACUCAUUCCUGA	*rip1-*siRNA-antisense	UCAGGAAUGAGUGCAUUUG
Scrambled-siRNA-sense	GAUCAUACGUGCGAUCAGA	Scrambled-siRNA-antisense	UCUGAUCGCACGUAUGAUC

### Caspase activity assays

Approximately 10^5^ ZF4 cells/mL were seeded in a 60-mm Petri dish (Nunc) and cultured for 24 h. The cells were infected with IPNV at a MOI of 5 and incubated for 6, 12, or 24 h at 18°C. Caspase-3, -8, or -9 activation assays [26] were performed using 10^6^ cells per timepoint. The cleavage of the Z-DEVD, Z-LETD, or Z-LEHD synthetic caspase-3, -8 and -9 substrates, respectively, was used to determine caspase activation using the Caspase-Glo Assay Kit (Promega). The assays were performed in 96-well plates and analyzed using a luminometer (VICTOR X2, PerkinElmer, USA). The amount of luminescence detected is directly proportional to the amount of caspase activation. All of the luminogenic substrate assay experiments were performed at the same time. Both the mock- and IPNV-infected caspase activation profiles were the same in all experiments and are included in each figure to facilitate comparisons. The results of all experiments are reported as the mean ± SEM.

### Intracellular ROS detection

Following infection with IPNV for the indicated time period (6, 12, 18 or 24 h), ZF4 cells were collected, washed twice with PBS, and incubated in warm HBSS/Ca/Mg solution containing 25 µM carboxy-H_2_DCFDA (Invitrogen) for 30 min at 37°C to detect ROS in live cells. ROS production was quantified using a fluorescent plate reader (VICTOR X2, PerkinElmer) with 485-nm emission and 525-nm absorption.

## Supporting Information

Figure S1 **IPNV infection of zebrafish embryonic cells (ZF4) induces host cell death.** (**A**) Detection of the viral protein expression profile in ZF4 cells following infection with IPNV (MOI=1) using western blot. The blot was probed using a polyclonal VP2-specific antibody, and lanes 1–4 correspond to 0, 6, 9, and 12 h p.i., respectively. The blot was probed with an actin-specific antibody as an internal control. (**B**) Viability of ZF4 cells infected with IPNV at an MOI of 1, 5, or 10 after 6, 12, 24, and 48 h. The viability for each sample was determined in three individual experiments. Data shown are the mean ± SD.
(DOC)

Figure S2 **Determination of gene expression levels at 0, 6, 12 and 24 h p.i. using quantitative real-time RT-PCR.** The expression profile of pro-apoptotic genes (**A**–**B**) were detected using quantitative real-time RT-PCR. The fold-change values of IPNV-infected cells compared to uninfected cells for genes representative of each of these groups is shown. The quantification of gene expression in IPNV-infected cells compared to uninfected control cells was calculated relative to the expression of *ef1a* as an internal control. Student's *t* tests indicate significant differences compared to 0 h: *, *p*<0.05; **, *p*<0.01.
(DOC)

Figure S3 **TNFα has the highest connectivity among the altered genes in microarray and quantitative RT-PCR experiments.** All of the altered genes were analyzed by Pathway Studio 6.0. The software is available from Ariadne Genomic Inc.
(DOC)

Table S1 **List of differentially expressed zebrafish mRNAs during IPNV infection identified using microarray analyses.** ^a^ Minus, decreased gene expression; no minus, increased gene expression; boldface, >2-fold-increased or decreased gene expression.
(DOC)

Table S2 **Comparison of the fold changes determined between microarray analysis and real-time PCR.** a) Quantitative real-time RT-PCR validation of oligo microarray data for genes that were up- or down-regulated in IPNV-infected versus uninfected control host cells at various timepoints post-infection. The genes validated using RT-PCR are also listed in Table S3. The quantification of gene expression in IPNV-infected versus uninfected control cells was done relative to the *ef1α* gene. b) Significant change in gene expression between IPNV-infected cells and uninfected cells as determined using microarray, *p*<0.05. c) Significant change in gene expression between IPNV-infected cells and uninfected cells as determined using quantitative real time RT-PCR, *p*<0.01.
(DOC)

Table S3 **Primer sequences for quantitative RT-PCR.**
(DOC)
