# Supplementary material for: Stage-Specific Expression of TNFα Regulates Bad/Bid-Mediated Apoptosis and RIP1/ROS-Mediated Secondary Necrosis in Birnavirus-Infected Fish Cells
Source: PLoS One. 2011 Feb 3;6(2):e16740. doi: 10.1371/journal.pone.0016740 (PMC3033425; doi:10.1371/journal.pone.0016740)
Supplement: Figure S1 — IPNV infection of zebrafish embryonic cells (ZF4) induces host cell death. (A) Detection of the viral protein expression profile in ZF4 cells following infection with IPNV (MOI=1) using western blot. The blot was probed using a polyclonal VP2-specific antibody, and lanes 1–4 correspond to 0, 6, 9, and 12 h p.i., respectively. The blot was probed with an actin-specific antibody as an internal control. (B) Viability of ZF4 cells infected with IPNV at an MOI of 1, 5, or 10 after 6, 12, 24, and 48 h. The viability for each sample was determined in three individual experiments. Data shown are the mean ± SD. (DOC) [file pone.0016740.s001.doc]

**Supplemental Material**

(A)


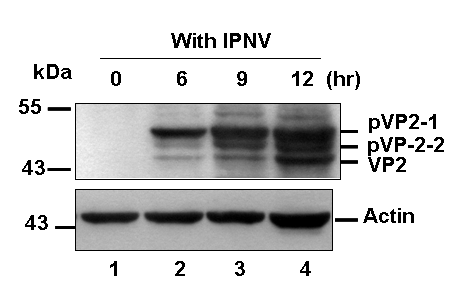


(B)

**Figure S1.** **IPNV infection of zebrafish embryonic cells (ZF4) induces host cell death.** (**A**) Detection of the viral protein expression profile in ZF4 cells following infection with IPNV (MOI=1) using western blot. The blot was probed using a polyclonal VP2-specific antibody, and lanes 1–4 correspond to 0, 6, 9, and 12 h p.i., respectively. The blot was probed with an actin-specific antibody as an internal control. (**B**) Viability of ZF4 cells infected with IPNV at an MOI of 1, 5, or 10 after 6, 12, 24, and 48 h. The viability for each sample was determined in three individual experiments. Data shown are the mean ± SD.
