# Supplementary material for: Stage-Specific Expression of TNFα Regulates Bad/Bid-Mediated Apoptosis and RIP1/ROS-Mediated Secondary Necrosis in Birnavirus-Infected Fish Cells
Source: PLoS One. 2011 Feb 3;6(2):e16740. doi: 10.1371/journal.pone.0016740 (PMC3033425; doi:10.1371/journal.pone.0016740)
Supplement: Figure S2 — Determination of gene expression levels at 0, 6, 12 and 24 h p.i. using quantitative real-time RT-PCR. The expression profile of pro-apoptotic genes (A–B) were detected using quantitative real-time RT-PCR. The fold-change values of IPNV-infected cells compared to uninfected cells for genes representative of each of these groups is shown. The quantification of gene expression in IPNV-infected cells compared to uninfected control cells was calculated relative to the expression of ef1a as an internal control. Student's t tests indicate significant differences compared to 0 h: *, p<0.05; **, p<0.01. (DOC) [file pone.0016740.s002.doc]

**Supplemental Material**

(A)

(B)

**Figure S2. Determination of gene expression levels at 0, 6, 12 and 24 h p.i. using quantitative real-time RT-PCR.** The expression profile of pro-apoptotic genes (**A**–**B**) were detected using quantitative real-time RT-PCR. The fold-change values of IPNV-infected cells compared to uninfected cells for genes representative of each of these groups is shown. The quantification of gene expression in IPNV-infected cells compared to uninfected control cells was calculated relative to the expression of *ef1a* as an internal control. Student’s *t* tests indicate significant differences compared to 0 h: *, *p* < 0.05; **, *p* < 0.01.
