# Supplementary material for: Stage-Specific Expression of TNFα Regulates Bad/Bid-Mediated Apoptosis and RIP1/ROS-Mediated Secondary Necrosis in Birnavirus-Infected Fish Cells
Source: PLoS One. 2011 Feb 3;6(2):e16740. doi: 10.1371/journal.pone.0016740 (PMC3033425; doi:10.1371/journal.pone.0016740)
Supplement: Figure S3 — TNFα has the highest connectivity among the altered genes in microarray and quantitative RT-PCR experiments. All of the altered genes were analyzed by Pathway Studio 6.0. The software is available from Ariadne Genomic Inc. (DOC) [file pone.0016740.s003.doc]

**Supplemental Material**


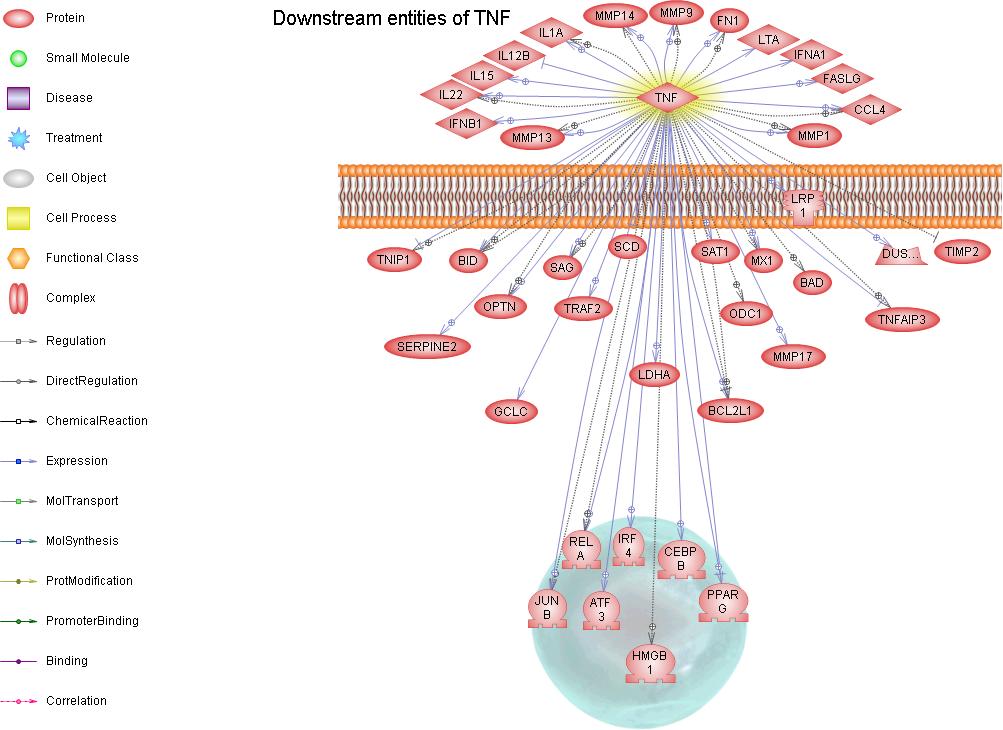


**Entities Downstream of TNFα**

**Figure S3. TNFα has the highest connectivity among the altered genes in microarray and quantitative RT-PCR experiments.** All of the altered genes were analyzed by Pathway Studio 6.0. The software is available from Ariadne Genomic Inc.
