# Supplementary material for: Stage-Specific Expression of TNFα Regulates Bad/Bid-Mediated Apoptosis and RIP1/ROS-Mediated Secondary Necrosis in Birnavirus-Infected Fish Cells
Source: PLoS One. 2011 Feb 3;6(2):e16740. doi: 10.1371/journal.pone.0016740 (PMC3033425; doi:10.1371/journal.pone.0016740)
Supplement: Table S1 — List of differentially expressed zebrafish mRNAs during IPNV infection identified using microarray analyses. a Minus, decreased gene expression; no minus, increased gene expression; boldface, >2-fold-increased or decreased gene expression. (DOC) [file pone.0016740.s004.doc]

**Table S1. List of differentially expressed zebrafish mRNAs during** IPNV infection identified using microarray analyses.

|  |  | Fold induction^a^ | | |  |  |
| --- | --- | --- | --- | --- | --- | --- |
| Gene Name | Symbol | 6 h | 12 h | 24 h | Related function(s) | GeneBank no. |
| Immune response |  |  |  |  |  |  |
| matrix metalloproteinase 9 | mmp9 | **8.3** | **14.8** | **9.43** | macrophage differentiation, positive regulation of apoptosis, extracellular matrix organization and biogenesis | NM _213123.1 |
| isgf-3 gamma | isgf3g | 1.07 | **3.43** | **3.47** | immune response, cell surface receptor linked signal transduction, regulation of transcription | NM_205710.1 |
| Tumor necrosis factor alpha | tnfa | **5.28** | **7.96** | **9.96** | immune response, regulation of apoptosis | BC156066 |
| TNFAIP3 interacting protein 1 | tnip1 | 1.74 | 1.09 | **2.84** | negative regulation of viral genome replication, defense response | BC083289.1 |
| tumor necrosis factor receptor associate factor-2 | traf2 | 0.84 | **2.05** | 1.74 | regulation of immunoglobulin secretion, regulation of apoptosis, regulation of JNK cascade | BX294186.9 |
| natural killer enhancing factor | nkef | 0.95 | **2.41** | **4.37** | cellular defense response | NM _ 001013471.1 |
| MHC class II integral membrane protein alpha chain | a2 | -1.3 | **-2.21** | **-2.17** | immune response | NM _ 131694 |
| Annexin a1 | anxa1a | -1.25 | -1.52 | **-2.09** | anti-inflammatory response | NM_181758.1 |
| Apoptosis |  |  |  |  |  |  |
| Requiem,apoptosis response zinc finger gene | req | **3.0** | **4.35** | **3.05** | induction of apoptosis by extracellular signals, Ubiquitin ligases | NM _ 212696.2 |
| bcl2-like | bcl2l | **2.38** | 1.96 | **2.55** | regulation of apoptosis, response to oxidative stress | NM _ 131807.1 |
| xiap, BIRC4 | xiap | 0.93 | 1.01 | **2.07** | negative regulation of apoptosis and caspase activity | AF439767.1 |
| Caspase 3, apoptosis-related cysteine protease | casp3 | 1.71 | **2.00** | 1.27 | positive regulation of apoptosis | NM _ 131877.2 |
| death associated protein 1b | dap1b | 1.62 | **2.32** | 1.62 | induction of apoptosis by extracellular signals | AF231128.1 |
| Transcription |  |  |  |  |  |  |
| Splicing factor u2af | u2af1 | **2.74** | **2.93** | **3.99** | RNA splicing | NM _ 205689.1 |
| ccaat/enhancer binding protein, beta | cebpb | **2.7** | **2.71** | **2.75** | induction of apoptosis, regulation of interleukin-6 biosynthesis, regulation of transcription, inflammatory response | NM _ 131884.2 |
| basic leucine zipper and W2 domains 1 | bzw1l | **2.57** | 1.6 | 1.31 | transcription antitermination, regulation of transcription, DNA-dependent | NM_213092.1 |
| core binding factor b subunit | cbfb | **2.56** | **2.24** | **2.26** | core-binding factor complex, transcription from RNA polymerase II promoter | NM_199209.1 |
| PHD finger protein 20 | phf20 | **2.3** | 1.13 | — | regulation of transcription, DNA-dependent | NM_001004114.1 |
| bromodomain and PHD finger containing | brpf1 | **2.28** | 1.4 | 1.32 | regulation of transcription, DNA-dependent | NM_201016.1 |
| creb-binding protein | crebbp | **2.2** | 1.52 | 1.39 | regulation of transcription, histone acetylation | BX901976.22 |
| cer-d4 xz isoform | cred4 | **2.2** | 1.44 | 1.33 | regulation of transcription, DNA-dependent | XM_686113.1 |
| DiGeorge syndrome critical region gene 6 | dgcrk6 | **2.18** | **2.53** | **2.58** | primary miRNA processing | BX005447.8 |
| coactivator-associated arginine methyltransferase 1 | carm1 | **2.11** | — | — | regulation of transcription, DNA-dependent | NM_001003645.1 |
| core promoter element binding protein | copeb | **2.11** | 1.48 | 1.46 | regulation of transcription, DNA-dependent | NM_201461.1 |
| yy1-associated factor 2 | yaf2 | **2.08** | 1.17 | 1.17 | regulation of transcription | AL929504.9 |
| polypyrimidine tract-binding protein 1 | ptb1 | 1.71 | **2.26** | **2.19** | mRNA processing, nuclear mRNA splicing | XM_704106.1 |
| chromodomain helicase DNA binding protein 6 | chd6 | — | **2.13** | — | regulation of transcription, chromatin remodeling | XM_694308.1 |
| RNA-binding protein NOB1 | nob1 | 1.71 | 1.89 | **2.01** | maturation of SSU-rRNA from tricistronic rRNA transcript , ribosomal small subunit biogenesis and assembly, | BC056558.1 |
| U6 snRNA-associated Sm-like protein LSm8 | lsm8 | 1.15 | 1.77 | **2.07** | mRNA metabolism, mRNA processing, nuclear mRNA splicing | XM_689282.1 |
| upstream binding transcription factor | ubtf | 1.99 | **2.43** | 1.8 | regulation of transcription, DNA-dependent | NM_201003.1 |
| Table S1 - Continued |  |  |  |  |  |  |
|  |  | Fold induction^a^ | | |  |  |
| Gene Name | Symbol | 6 h | 12 h | 24 h | Related function(s) | GeneBank no. |
| DAZ associated protein 2 | dazap2 | 1.26 | **2.07** | **2.15** | transcription factor complex | NM_199793.2 |
| neurogenin 1 | neurog1 | 1.99 | **3.13** | **2.91** | neuron differentiation, regulation of transcription, DNA-dependent | NM_131041.1 |
| v-maf musculoaponeurotic fibrosarcoma oncogene family protein B | mafb | **2.11** | 1.16 | — | regulation of transcription, DNA-dependent | NM_131015_1 |
| high-mobility group box 1 | hmgb1 | 1.43 | **2.63** | **2.82** | regulation of transcription, DNA repair, DNA recombination | NM_199555.2 |
| cleavage and polyadenylation specific factor 1 | cpsf1 | 1.34 | **2.45** | 1.91 | mRNA polyadenylation, mRNA cleavage | BC090495.1 |
| proteasome 26S subunit, non-ATPase (Grakyrin) | psmd10 | 1.0 | **2.49** | **2.61** | regulation of transcription, DNA-dependent, regulation of cell proliferation, regulation of apoptosis | NM_205754.1 |
| tumor protein p53 | tp53 | **-2.02** | -1.63 | -1.51 | regulation of progression through cell cycle, G1 DNA damage checkpoint, induction of apoptosis , regulation of cell proliferation, regulation of transcription, DNA-dependent | NM_131327.1 |
| homeo box B4a | hoxb4a | **-2.25** | -1.83 | -1.87 | regulation of transcription, organ morphogenesis | NM_131118_1 |
| hypoxia-inducible factor 1, alpha subunit inhibitor | hif1an | **-2.13** | -1.75 | -0.98 | regulation of transcription, DNA-dependent, oxidoreductase activity | NM_201496.1 |
| runt-related transcription factor 1 | runx1 | **-2.92** | -0.93 | -1.57 | regulation of transcription, DNA-dependent | NM_131603.2 |
| transcription factor pitx2a | pitx2a | **-2.27** | -1.65 | -1.48 | transcription termination, regulation of transcription, DNA-dependent | NM_130975 |
| muscle segment homeobox a | msx1 | **-3.62** | **-2.58** | -1.6 | regulation of transcription, negative regulation of transcription | BC095602.1 |
| ladybird homeobox homolog 1 | lbx1 | **-3.86** | **-2.7** | **-2.36** | regulation of transcription | NM_001007134.1 |
| inhibitor of DNA binding 1 | id1 | **-5.1** | **-2.53** | **-2.02** | regulation of transcription, regulation of angiogenesis | NM_131245.1 |
| signal transduction and activation of transcription 3 | stat3 | -1.34 | -1.39 | **-2.02** | regulation of transcription, DNA-dependent | NM_131479.1 |
| v-jun sarcoma virus 17 oncogene | jun | -1.37 | **-2.42** | **-2.67** | regulation of progression through cell cycle, regulation of transcription, DNA-dependent | NM_199987.1 |
| c-myc | myc | -0.86 | **-2.32** | -1.33 | regulation of transcription, DNA-dependent | NM_200172.1 |
| SWI/SNF related, matrix associated, actin dependent regulator of chromatin | smarca1 | -1.03 | -1.99 | **-2.23** | regulation of transcription, DNA-dependent | NM_212716.1 |
| rna binding motif protein 5 | rbm5 | **-2.22** | -1.27 | -1.34 | RNA processing | NM_001100138 |
| methyl-CpG binding domain protein 3b | mbd3b | **-2.27** | **-2.27** | **-3.95** | histone acetylation, regulation of transcription | NM_212580.1 |
| Signal transduction |  |  |  |  |  |  |
| arrestin | arrdc2 | **5.76** | **3.16** | **2.65** | response to stimulus, signal transduction | CR450818.8 |
| mitogen-activated protein kinase 6 | erk3 | **3.9** | **2.22** | **2.54** | protein serine/threonine kinase activity, transferase activity | AL935189.7 |
| dual specificity phosphatase 6 | dusp6 | **3.12** | 0.83 | 0.79 | regulation of progression through cell cycle, inactivation of MAPK activity | NM_194380.1 |
| SH3-binding domain protein 5-like | sh3bp5l | **2.47** | 1.34 | 1.21 | signal transduction | NM_213160.1 |
| serum/glucocorticoid regulated kinase | sgk | **2.36** | 1.15 | 1.46 | protein amino acid phosphorylation, response to DNA damage stimulus and stress | NM_199212.1 |
| GTP binding protein 1 | gtpbp1 | **2.29** | 1.62 | **2.38** | electron carrier activity, immune response, Gtp binding proteins | NM_213475.1 |
| GTP binding protein 4 | gtpbp4 | **2.08** | 1.27 | 1.67 | small GTPase mediated signal transduction | NM_199851.1 |
| suppressors of cytokine signaling 3 | socs3 | 1.88 | **2.39** | **3.15** | intracellular signaling cascade, regulation of cell growth | NM_199950.1 |
| G-protein signaling modulator 2 | gpsm2 | 1.85 | **2.54** | 1.97 | GTPase activator activity, G-protein coupled receptor protein signaling pathway | NM_200438.1 |
| leucine zipper-ef-hand containing transmembrane protein 1 | letm1 | **2.24** | **2.01** | 1.76 | signal transduction | XM_694258.1 |
| Table S1 - Continued |  |  |  |  |  |  |
|  |  | Fold induction^a^ | | |  |  |
| Gene Name | Symbol | 6 h | 12 h | 24 h | Related function(s) | GeneBank no. |
| low-density lipoprotein receptor-related protein 1 | lrp1 | **2.03** | 1.66 | 1.27 | negative regulation of Wnt receptor signaling pathway, endocytosis | XM_689066.1 |
| attractin-like protein | atrnl1 | **2.07** | 1.22 | 0.92 | G-protein coupled receptor protein signaling pathway | XM_692563.1 |
| Heme oxygenase 2 | hmox2 | **-9.01** | **-7.35** | **-3.95** | electron carrier activity, oxidoreductase activity | XM_690455.1 |
| phosphatase and actin regulator 4 | phactr4 | **-2.13** | -0.94 | -1.02 | aromatic compound metabolism, proteolysis | NM_200670.1 |
| platelet-derived growth factor receptor alpha | pdgfra | **-3.19** | **-2.24** | -1.76 | protein serine/threonine kinase | NM_131459.2 |
| Down syndrome candidate region 1-like protein 1 | dscr1l1 | **-2.39** | **-2.43** | **-2.16** | calcium-mediated signaling | NM_214819.1 |
| calmodulin 2, delta;calmodulin 2 | calm2d | -1.38 | -1.86 | **-2.11** | G protein coupled receptor protein signaling pathway | NM_199996.2 |
| protein disulfide isomerase-related protein 5 | pdip5 | -1.48 | -1.87 | **-2.14** | negative regulation of signal transduction | NM_197933.2 |
| Lipid and cholesterol metabolism |  |  |  |  |  |  |
| acyl-CoA synthetase long-chain family member 4 | acsl4 | **3.34** | **2.0** | **2.83** | Fatty acid metabolism, lipid metabolism | NM_200649.1 |
| long chain acyl-coa synthetase | lacs | **2.95** | 1.64 | **2.40** | Fatty acid metabolism, long-chain-fatty-acid-CoA ligase activity | BX088535.6 |
| stearoyl-CoA desaturase | scd | **2.3** | 1.44 | 1.57 | lipid and fatty acid biosynthesis, fatty acid desaturation | NM_198815.1 |
| long-chain fatty-acyl elongase | lce | **2.11** | **3.93** | **2.29** | fatty acid elongation, transferring groups other than amino-acyl groups | NM_199532.1 |
| phenylalkylamine Ca2+ antagonist binding protein | ebp | **-2.02** | **-2.17** | -1.25 | sterol metabolism, cholesterol biosynthesis | NM_001002328.1 |
| 3-hydroxy-3-methylglutaryl-coenzyme a reductase | hmgcr | **-2.03** | -1.52 | -1.46 | cholesterol biosynthesis, steroid biosynthesis | BX511209.11 |
| hydroxyacyl-Coenzyme A dehydrogenase | hadh | **-2.27** | -1.44 | -1.34 | lipid metabolism | NM_001003515.1 |
| lipoprotein lipase | lpl | **-2.79** | **-3.82** | **-2.26** | triacylglycerol biosynthesis, fatty acid metabolism, lipid metabolism | NM_131127.1 |
| ATP citrate lyase | acly | -1.0 | -1.53 | **-2.02** | lipid synthesis, ATP catabolism | NM_001002649.1 |
| oxysterol binding protein-like 2 | osbpl2 | -1.13 | **-2.08** | -1.27 | sterol metabolism, lipid transport | NM_199872.1 |
| farnesyl diphosphate synthase | fdps | **-2.5** | **-2.32** | -1.99 | cholesterol biosynthesis, steroid biosynthesis | NM_001025471.1 |
| nadph steroid dehydrogenase | nsdhl | -1.68 | **-2.12** | -1.83 | steroid synthesis | NM_001017674.1 |
| Carbohydrate metabolism |  |  |  |  |  |  |
| fructose-6-phosphate 2-kinase | fkfbp | **2.17** | 1.16 | — | fructose 2,6-bisphosphate metabolic process | NM_213397.1 |
| UDP-glucose dehydrogenase | ugdh | **2.47** | **11.79** | **11.77** | UDP-glucose metabolism, UDP-glucuronate biosynthesis, electron carrier activity | NM_131852.1 |
| lactate dehydrogenase A4 | ldha | 1.55 | **2.39** | **2.93** | tricarboxylic acid cycle intermediate metabolism, glycolysis | NM_131246_1 |
| meningioma expressed antigen 5 | mgea5 | **2.21** | **2.01** | 1.97 | glycoprotein catabolism, hyaluronoglucosaminidase activity | XM_695280.1 |
| transaldolase 1 | taldo1 | **-2.33** | **-2.19** | — | carbohydrate metabolism, transaldolase activity | BC046015.1 |
| Oxidative phosphorylation |  |  |  |  |  |  |
| ATPase | atp2a2 | **2.01** | 1.29 | 1.12 | ion transport, proton transport, ER-nuclear signaling pathway, | NM_200965.1 |
| ATPase family, AAA domain containing 2 | pro2000 | 1.6 | **2.63** | **2.13** | nucleoside-triphosphatase activity | XM_690657.1 |
| diaphorase (NADH) (cytochrome b-5 reductase) | dia1 | 1.09 | 1.57 | **2.01** | electron transport | NM_200189.2 |
| aldehyde dehydrogenase 2 | ALDH2 | -1.38 | -1.93 | **-2.07** | electron carrier activity, carbohydrate metabolism, alcohol metabolism | NM_213301.2 |
| cytochrome b | cytb | -1.45 | -1.36 | **-2.29** | electron transport | AC024175.3 |
| Cell cycle |  |  |  |  |  |  |
| G1 to S phase transition 1 | gspt1 | **2.33** | 1.45 | 1.29 | G1-S transition of mitotic cell cycle, regulation of apoptosis | NM_198806.1 |
| protein kinase; chk1 | chk1 | **2.33** | 1.51 | 1.36 | DNA damage checkpoint, protein amino acid phosphorylation | NM_200193.1 |
| CDC23 | cdc23 | 1.79 | **2.49** | **2.32** | regulation of mitotic metaphase-anaphase transition, ubiquitin-dependent protein catabolism | NM_200933.1 |
| Table S1 - Continued |  |  |  |  |  |  |
|  |  | Fold induction^a^ | | |  |  |
| Gene Name | Symbol | 6 h | 12 h | 24 h | Related function(s) | GeneBank no. |
| cyclin D1 | ccnd1 | **-2.6** | **-2.1** | -1.53 | regulation of progression through cell cycle | NM_131025.2 |
| MBF transcription factor complex subunit Cdc10 | cdc10 | 1.39 | 1.44 | **2.0** | G1/S-specific transcription in mitotic cell cycle, cytokinesis | NM_201161.1 |
| Protein degradation |  |  |  |  |  |  |
| ornithine decarboxylase | Odc1 | **4.62** | — | — | positive regulation of cell proliferation, ornithine decarboxylase activity | NM_131801.2 |
| polyubiquitin | ubb | **3.66** | 1.64 | 1.38 | ATP-dependent degradation of denatured proteins | BX323825.19 |
| Nitric oxide synthase interacting protein | nosip | **2.13** | 1.35 | 1.28 | protein ubiquitination | NM_001007434.1 |
| proteasome activator subunit 2 | psme2 | 0.96 | **2.95** | **2.71** | proteasome activator activity | NM_131374.1 |
| cathepsin h | ctsh | 1.17 | **2.0** | **2.7** | cysteine-type peptidase activity, proteolysis | NM_212688.1 |
| abhydrolase domain containing 4 | abhd4 | 1.86 | **2.2** | **2.12** | aromatic hydrocarbon metabolism, proteolysis | NM_001017613.1 |
| proteasome 26S subunit, ATPase, 1b | psmc1b | 1.36 | 1.76 | **2.25** | protein degradation | NM_001002091.1 |
| nonspecific cytotoxic cell receptor protein 1 | nccrp1 | 1.39 | 1.9 | **2.08** | protein degradation | NM_130921.1 |
| ubiquitin fusion degradation 1-like | ufd1l | 1.16 | 1.87 | **2.1** | ubiquitin-dependent protein catabolism | NM_001002451.1 |
| endothelin converting enzyme-1 | ece1 | 0.98 | 1.56 | **2.66** | proteolysis, peptide hormone processing | XM_689595.1 |
| glutaminyl-peptide cyclotransferase-like | qpctl | **2.26** | — | — | proteolysis | XM_684546.1 |
| F-box only protein 30 | fbxo30 | -1.47 | -1.79 | **-2.19** | ubiquitin cycle | NM_201151.1 |
| Protein folding and stress response |  |  |  |  |  |  |
| heat shock 70-kDa protein 5 (Grp78) | hspa5 | **2.05** | 0.91 | 0.89 | response to unfolded protein, negative regulation of caspase activity | NM_213058.1 |
| 90-kda heat shock protein | hsp90 | **-2.18** | **-2.67** | -1.34 | response to unfolded protein, mitochondrial transport | CR381646.8 |
| DnaJ (Hsp40) homolog | dnaja1l | **-2.2** | -1.42 | -0.91 | unfolded protein binding | NM_199662.1 |
| heat shock protein 47 | hsp47 | **-11.76** | **-10.55** | **-5.05** | serine-type endopeptidase inhibitor activity | NM_131204.1 |
| tumor rejection antigen (gp96) 1 | tra1 | -1.59 | **-3.57** | **-3.99** | response to unfolded protein, negative regulation of caspase activity | NM_198210.2 |
| Protein synthesis |  |  |  |  |  |  |
| glutamate-ammonia ligase b | glulb | **2.37** | 1.89 | 1.51 | glutamine biosynthetic process | NM_182866.1 |
| ribosomal protein L8 | rpl8 | **2.18** | 1.19 | 1.34 | protein biosynthesis | BX005425.12 |
| cold inducible RNA binding protein | cirbp | **2.13** | **2.93** | **4.54** | eukaryotic translation elongation factor 1 complex | NM_200017.1 |
| eukaryotic translation initiation factor 4A | eif4a1a | **2.11** | 1.12 | 1.53 | ATP-dependent helicase activity, response to zinc ion, induction of apoptosis | NM_198366.1 |
| glutamate-cysteine ligase | gclm | 1.72 | **2** | **2.08** | glutamate-cysteine ligase activity | NM_199845.1 |
| mitochondrial ribosomal protein L3 | mrpl3 | 1.53 | **2.5** | 1.62 | mitochondrial large ribosomal subunit, protein biosynthesis | NM_200007.1 |
| eukaryotic translation initiation factor 4 gamma | elf4g | **-2.28** | **-3.6** | **-4.35** | regulation of translational initiation | XM_691617.1 |
| eukaryotic translation initiation factor 2, subunit 1 alpha | eif2s1 | -1.2 | -1.75 | **-2.27** | regulation of translation, regulation of translation initiation in response to stress | NM_131800.2 |
| 5-methyltetrahydrofolate-homocysteine methyltransferase | mtr | -1.38 | **-2.13** | -1.66 | amino acid biosynthesis, methyltransferase activity | NM_198072.1 |
| pseudouridine synthase 1 | pus1 | -1.33 | **-2.14** | -1.72 | tRNA processing | NM_001008603.1 |
| tyrosyl-tRNA synthetase | yars | -1.48 | **-2.26** | -1.73 | tRNA aminoacylation for protein translation | NM_201316.1 |
| Nucleoside metabolism and synthesis |  |  |  |  |  |  |
| thymidine kinase 2 | tk2 | **2.22** | 1.72 | 1.67 | DNA replication, nucleobase, nucleoside, nucleotide and nucleic acid metabolism | NM_001002743.1 |
| Table S1 - Continued |  |  |  |  |  |  |
|  |  | Fold induction^a^ | | |  |  |
| Gene Name | Symbol | 6 h | 12 h | 24 h | Related function(s) | GeneBank no. |
| polymerase (DNA directed), epsilon 2 | pole2 | 1.32 | **4.69** | **4.01** | DNA replication | NM_173246.1 |
| 5-nucleotidase, cytosolic IA | nt5c1a | — | 1.46 | **2.07** | nucleoside metabolism | XM_685092.1 |
| chromobox homolog 1 | cbx1 | -1.89 | **-2.27** | **-2.27** | chromatin assembly or disassembly | NM_199746.1 |
| dUTP pyrophosphatase | dut | — | **-2.36** | **-2.27** | DNA replication, dUTP metabolism | NM_001006005.1 |
| proliferating cell nuclear antigen | pcna | -1.5 | **-2.43** | -1.32 | DNA replication, regulation of DNA replication | NM_131404.1 |
| primase polypeptide 1 | prim1 | **-2.11** | -1.45 | -1.22 | DNA replication, synthesis of RNA primer | NM_201448.1 |
| adenosine kinase a | adka | -1.18 | -1.52 | **-2.14** | ribonucleoside monophosphate synthesis, purine ribonucleoside salvage | NM_212791.1 |
| SET domain-containing protein 7 | setd7 | -1.07 | -1.94 | **-2.66** | chromatin modification | NM_001002456.1 |

^a^ Minus, decreased gene expression; no minus, increased gene expression; boldface, >2-fold–increased or decreased gene expression.
