# Supplementary material for: Stage-Specific Expression of TNFα Regulates Bad/Bid-Mediated Apoptosis and RIP1/ROS-Mediated Secondary Necrosis in Birnavirus-Infected Fish Cells
Source: PLoS One. 2011 Feb 3;6(2):e16740. doi: 10.1371/journal.pone.0016740 (PMC3033425; doi:10.1371/journal.pone.0016740)
Supplement: Table S2 — Comparison of the fold changes determined between microarray analysis and real-time PCR. a) Quantitative real-time RT-PCR validation of oligo microarray data for genes that were up- or down-regulated in IPNV-infected versus uninfected control host cells at various timepoints post-infection. The genes validated using RT-PCR are also listed in Table S3. The quantification of gene expression in IPNV-infected versus uninfected control cells was done relative to the ef1α gene. b) Significant change in gene expression between IPNV-infected cells and uninfected cells as determined using microarray, p<0.05. c) Significant change in gene expression between IPNV-infected cells and uninfected cells as determined using quantitative real time RT-PCR, p<0.01. (DOC) [file pone.0016740.s005.doc]

**Table S2. Comparison of the fold changes determined between microarray analysis and real-time PCR. ^(a)^**

| **Gene** | **Symbol** | **Time**  **(h p.i.)** | **Micro-**  **Arrays (b)** | **Q-PCR (c)** |
| --- | --- | --- | --- | --- |
| matrix metalloproteinase 9 | mmp9 | 6 h | 8.3* | 8.29 ^#^ |
|  |  | 12 h | 14.8* | 20.69 ^#^ |
|  |  | 24 h | 9.43 * | 8.33 ^#^ |
| isgf-3 gamma | isgf3g | 6 h | 1.07 | 1.32 |
|  |  | 12 h | 3.43 * | 3.5 ^#^ |
|  |  | 24 h | 3.47 * | 2.3 ^#^ |
| Tumor necrosis factor alpha | tnfa | 6 h | 5.28 * | 4.73 ^#^ |
|  |  | 12 h | 7.96 * | 6.67 ^#^ |
|  |  | 24 h | 9.96 * | 9.43 ^#^ |
| bcl2-like | bclxl | 6 h | 2.38 * | 5.87 ^#^ |
|  |  | 12 h | 1.96 * | 5.51 ^#^ |
|  |  | 24 h | 2.55 * | 6.39 ^#^ |
| ccaat/enhancer binding protein, beta | cebpb | 6 h | 2.7 * | 3.9 ^#^ |
|  |  | 12 h | 2.7 * | 3.94 ^#^ |
|  |  | 24 h | 2.75 * | 3.08 ^#^ |
| lipoprotein lipase | lpl | 6 h | -2.79 * | -2.75 ^#^ |
|  |  | 12 h | -3.82 * | -5.16 ^#^ |
|  |  | 24 h | -2.26 * | -3.11 ^#^ |
| v-jun sarcoma virus 17 oncogene | jun | 6 h | -1.37 | -1.87 ^#^ |
|  |  | 12 h | -2.42 * | -2.18 ^#^ |
|  |  | 24 h | -2.67 * | -2.29 ^#^ |
| Heat shock protein 47 | hsp47 | 6 h | -11.71 * | -7.69 ^#^ |
|  |  | 12 h | -10.55 * | -16.76 ^#^ |
|  |  | 24 h | -5.05 * | -7.06 ^#^ |

a) Quantitative real-time RT-PCR validation of oligo microarray data for genes that were up- or down-regulated in IPNV-infected versus uninfected control host cells at various timepoints post-infection. The genes validated using RT-PCR are also listed in Table 1. The quantification of gene expression in IPNV-infected versus uninfected control cells was done relative to the EF1α gene.

b) Significant change in gene expression between IPNV-infected cells and uninfected cells as determined using microarray, *p*<0.05.

c) Significant change in gene expression between IPNV-infected cells and uninfected cells as determined using quantitative real time RT-PCR, *p*<0.01.
