# Supplementary material for: Stage-Specific Expression of TNFα Regulates Bad/Bid-Mediated Apoptosis and RIP1/ROS-Mediated Secondary Necrosis in Birnavirus-Infected Fish Cells
Source: PLoS One. 2011 Feb 3;6(2):e16740. doi: 10.1371/journal.pone.0016740 (PMC3033425; doi:10.1371/journal.pone.0016740)
Supplement: Table S3 — Primer sequences for quantitative RT-PCR. (DOC) [file pone.0016740.s006.doc]

**Table S3. Primer sequences for quantitative RT-PCR.**

| **Oligo Name** | **Sequence 5'→3'** | **Oligo Name** | **Sequence 5'→ 3'** |
| --- | --- | --- | --- |
| *bmf1*-5p | CCCACCTGCAGATAAAGCAGA | *bmf1*-3p | CCGTAGAGAAAGTGTCCGTGC |
| *bmf2*-5p | ATGGAAACGCTGGATTTCGT | *bmf2*-3p | TCTGGTCTGCCTTCGTCCTG |
| *noxa*-5p | AGAGCAAACCGCTGTAGTAGAGTG | *noxa*-3p | TTGTATTTTCTGGAGTGTTACTATGAGCT |
| *puma*-5p | GACCCCACACTGGAGGAGC | *puma*-3p | GGGACGGCATTCCTCTGAA |
| *bax*-5p | AGGGTGGATGGGACGGAAT | *bax*-3p | TTGCGAATCACCAATGCTGT |
| *bax2*-5p | GCTCGCCTACCCAGAATTGTT | *bax2*-3p | TTCATCCATCCTCCTACTGTGAGA |
| *bok1*-5p | ATCAAATCCAGGCCTCGTCC | *bok1*-3p | TGTTGCGATGATGTCACATAATG |
| *bok2*-5p | CAGACAAACATGCGGGACAT | *bok2*-3p | GACGAGCTCTTTCTCCGTGTG |
| *bid*-5p | ATGGGACAGTGGTGCAGTTTT | *bid*-3p | AATCTTTCACTTCTCTAACTGCTCAACA |
| *bad*-5p | AGTGGGTCGGCACAGAAAAA | *bad*-3p | TGAAAGGATCCCCAAGCATATG |
| *tnfa*-5p | AGGACCAGGCCTTTTCTTCAG | *tnfa* -3p | AATGCCATCATCGGGAATGA |
| *rip1*-5p | GATCTCTCGGCTTGTAGCATGA | *rip1*-3p | ATATGTACAGAGCAGGGAGAAAACTAACA |
| *ef1a*-5p | TGCCTTCGTCCCAATTTCAG | *ef1a* -3p | TACCCTCCTTGCGCTCAATC |
